# Lockdown logistics in consultation–liaison psychiatry

**DOI:** 10.1017/ipm.2020.104

**Published:** 2020-09-11

**Authors:** H. Barry, A. M. Doherty, M. Clancy, S. Moore, S. MacHale

**Affiliations:** 1 Department of Liaison Psychiatry, Beaumont University Hospital, Dublin, Ireland; 2 Department of Psychiatry, University Hospital Galway, Galway, Ireland; 3 Department of Psychiatry, University Hospital Waterford, Waterford, Ireland; 4 Department of Psychiatry, St Vincent’s University Hospital, Dublin, Ireland

**Keywords:** COVID-19, psychiatry, liaison psychiatry

## Abstract

We describe the adaptation of services to allow flexible and practical responses to the coronavirus-19 (COVID-19) public health crisis by four Consultation–Liaison Psychiatry (CLP) services; Galway University Hospital (GUH), Beaumont Hospital, University Hospital Waterford and St Vincent’s University Hospital (SVUH) CLP services. This article also illustrates close collaboration with community adult mental health services and Emergency Department (ED) colleagues to implement effective community diversion pathways and develop safe, effective patient assessment pathways within the EDs. It highlights the high levels of activity within each of the CLP services, while also signposting that many of the rapidly implemented changes to our practice may herald improvements to mental health patient care delivery in the post-COVID-19 world, if our psychiatry services receive appropriate resources.

## Introduction

Coronavirus-19 (COVID-19) is a disease caused by the virus SARS-CoV-2, and following the initial outbreak in Wuhan, China, it spread to many other countries and was declared a pandemic by the World Health Organization on 12 March 2020. The emergence of COVID-19 has necessitated significant changes to the operation of Consultation–Liaison Psychiatry (CLP) services in order to continue to maintain a high standard of clinical care. New considerations include social distancing in the community and healthcare settings, and in acute hospitals, the avoidance of unnecessary exposure of patients and staff to COVID-19 to limit potential cross-contamination between our various patient cohorts, to minimise staff illness and to preserve PPE.

As for all healthcare services, COVID-19 has brought new challenges across all areas of CLP practice. Our inpatient work has altered in terms of the increased acuity of presentations in patients with serious mental disorders who have been too frightened to present to services until very unwell, infection-related neuropsychiatric presentations, and COVID-19 exacerbated deteriorations in mental health resulting in serious self-harm necessitating medical and surgical admissions, all in addition to the considerable work being undertaken nationally by CLP teams to assess and manage the mental health impact on front-line health workers of the current high-stress environment (Greenberg *et al*. 2020; Troyer *et al.* 2020).

For the purposes of this paper, we will focus on the changes made to the Emergency Department (ED) component of CLP practice in four different services across Ireland. CLP services work within a variety of governance frameworks nationally; some within the acute hospitals service, others funded by mental health services, and each has different staffing and environmental considerations, factors which have influenced the individual service changes. CLP services in EDs manage crisis presentations, which range from self-harm and suicidal ideation to relapse of severe mental illnesses such as psychosis and mood disorders and new-onset psychopathology (Yao *et al*. 2020). In addition, vulnerable populations such as people living in homelessness may seek urgent mental health care through EDs, and these individuals are potentially at even greater risk in the context of COVID-19 (Lima *et al*. 2020).

The need to protect patients and staff from further spread of COVID-19 has resulted in the integration of remote consulting technologies in psychiatry more widely, but also in CLP practice to reduce the duration of face-to-face contact (Wing *et al*. 2020; Whaibeh *et al*. 2020).

We describe four services which represent a range of responses to the crisis: Galway University Hospital (GUH), Beaumont Hospital, University Hospital Waterford and St Vincent’s University Hospital (SVUH) CLP services to provide a national perspective of a flexible, adaptable and practical specialty response by CLP to the current public health crisis.

## GUHs

GUHs is a 900-bed tertiary hospital in the west of Ireland, which is spread over two sites: the main University Hospital Galway (UHG) site includes the ED and most acute specialities and the Merlin Park site includes geriatric rehabilitation, elective orthopaedic surgery and day procedures including dialysis and infusion of biologic agents.

There is a 50-bedded Acute Adult Mental Health Unit (AAMHU) on the GUH site. The ED refers approximately 2000 patients to psychiatry (CLP team 9–5 p.m., on-call system out of hours) per annum and there are on average 1100-ward-based consultation requests resulting in 2500-ward-based psychiatric reviews. GUH has a small CLP Team, comprising 1 FTE consultant, 1 FTE NCHD and 3 FTE nurses (1 Advanced Nurse Practitioner and 2 Clinical Nurse Specialists (CNSs): one of whom is from the National Clinical Programme in Self-Harm): less than one-third of the minimum Vision for Change and PLAN standards (DOH, 2005; PLAN, 2014).

**Fig. 1.**
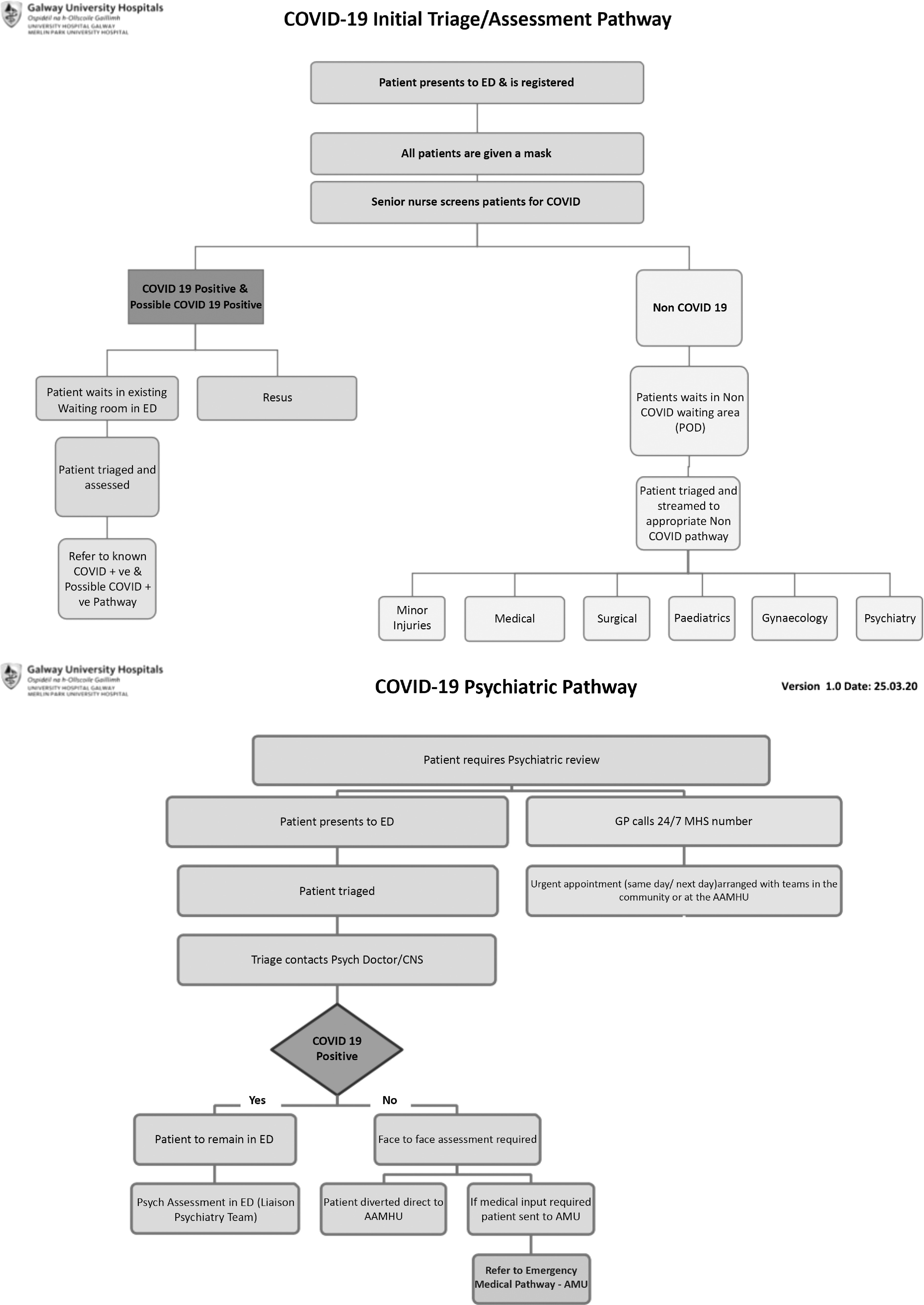
GUH assessment pathways for the duration of COVID-19.

The hospital has implemented significant changes to acute working to allow the separation of patients with confirmed or likely COVID-19 from other patients to limit spread. Local acute hospital planning for COVID-19 is influenced by the relatively small size of the ED at UHG: the ED is allocated as the COVID-19 pathway with presentations redirected from triage and the non-COVID-19 pathway involves assessment in the Acute Medical Unit (AMU) or emergency surgical ward, depending on the presenting complaint (see Fig. 1).

The Galway Roscommon Mental Health Service has implemented an emergency plan to reduce exposure of patients attending the service to infection by avoiding unnecessary hospital presentations and to optimise the care of patients seen in the community, consistent with the RCPsych COVID-19 liaison psychiatry guidelines (RCPsych, 2020). This has two components which are explained in the following.

## Community diversion

There is a new single telephone number (24/7) for the whole service, which is answered by a clinician and the call is diverted appropriately. Where possible, calls are forwarded to the appropriate Community Mental Health Team (CMHT), who make every effort to deal with the query or emergency in the community, either remotely or face to face as appropriate.

## Psychiatry emergency referral service (Psych ED)

Patients may be referred directly to this service in the AAMHU by their GP or from ED triage in the case of self-presentation. Previously, all patients presenting to the hospital for emergency assessment were reviewed in the ED. During normal working hours patients are reviewed by CLP, and out of hours by the registrar on call, and or the CNS on call which is a new rota for the duration of the COVID-19 emergency.

Thus, there are three pathways for acute referrals to CLP (Fig. 1).

### Psych ED (As detailed above where patients present to ED and are triaged directly to CLP)

#### Non-COVID-19 pathways

Patients with medical or surgical acute needs (including self-harm) are sent directly from triage to the AMU and the Acute Surgical Unit as appropriate, and are seen by CLP in parallel with their medical or surgical treatment either at these clinical decision units or on hospital wards including critical care, consistent with the National Guidelines (HSE, 2016).

#### The COVID-19 pathway

This is the pathway from ED to the designated COVID-19 wards, including critical care.

Providing a prompt service to three acute pathways is challenging for a small team. In 2 weeks since launch, it has been found to be workable. Using a quality improvement approach, small Plan–Do–Study–Act (PDSA) cycles have been applied to troubleshoot small issues that have arisen, these include minor difficulties such as how to physically transport patients from the ED triage area to the new Psych ED at the AAMHU, and the management of intoxicated persons in the AMU.

## Beaumont Hospital

Beaumont Hospital is a large academic teaching hospital 5 km north of Dublin City centre. It provides emergency and acute care services across 54 medical specialties to a local community of some 290 000 people, as well as being a National Centre for Neurosurgery, Cochlear Implant and Renal Transplantation. The hospital employs approximately 3000 staff and has 889 beds in total, with approximately 1200 ED mental health presentations, 1400 ward consults and 4 new and returning patient OPD clinics per week, per year. The CLP team comprises 2 WTE Liaison psychiatry consultants and a 0.5 WTE renal transplant consultant, 4 WTE NCHDs and currently 2 WTE Self-Harm CNSs. At the time of submission, Beaumont Hospital had the largest number of confirmed COVID-19 cases, 137 cases on 15 April, over a third more than any other hospital in the country.

Patients presenting to the Beaumont Hospital ED CLP team typically live within either the North Dublin Mental Health Service (NDMHS) or St Vincent’s Hospital Fairview North Central catchment area (SVF). Thirty-eight per cent of patients presenting to CLP are currently attending a CMHT (Beaumont Hospital June 2016–February 2020 Electronic Patient Record Dive System Report Data). There is no AAMHU within the Beaumont Hospital, but the NDMHS operates an autonomous 49-bed unit within the campus with no on-site emergency assessment facility. The SVF service offers acute and emergency mental health assessments in the St Vincent’s Hospital Fairview site, and typically only patients with comorbid acute medical needs present to Beaumont Hospital CLP from this service.

## Community diversion

As the COVID-19 situation emerged, consideration was given to redirecting a proportion of the 59% of the patients who present to the ED CLP team without an acute physical healthcare need (Beaumont Hospital Electronic Patient Record Dive System Report Data) to an alternative site out with the ED, staffed by the CLP team, but this was not feasible within the space resources available. The CMHTs worked to ensure that community patients were seen rapidly wherever possible and good communication between administrative support workers and GPs was seen as essential in this regard.

The NDMHS operates a rapid access number which is answered by an administrative staff member who then directs the query to the appropriate catchment area team.

## ED pathway

**Fig. 2.**
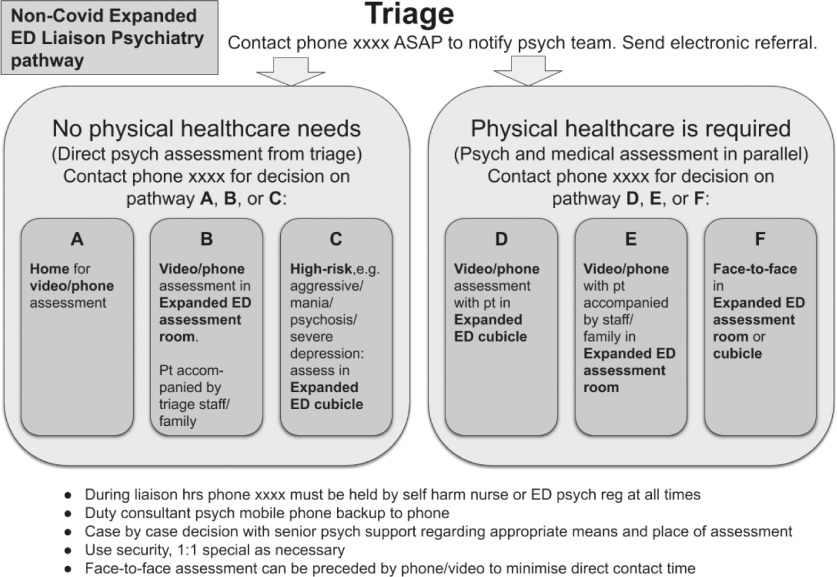
Beaumont Hospital non-COVID-19 ED patient algorithm.

**Fig. 3.**
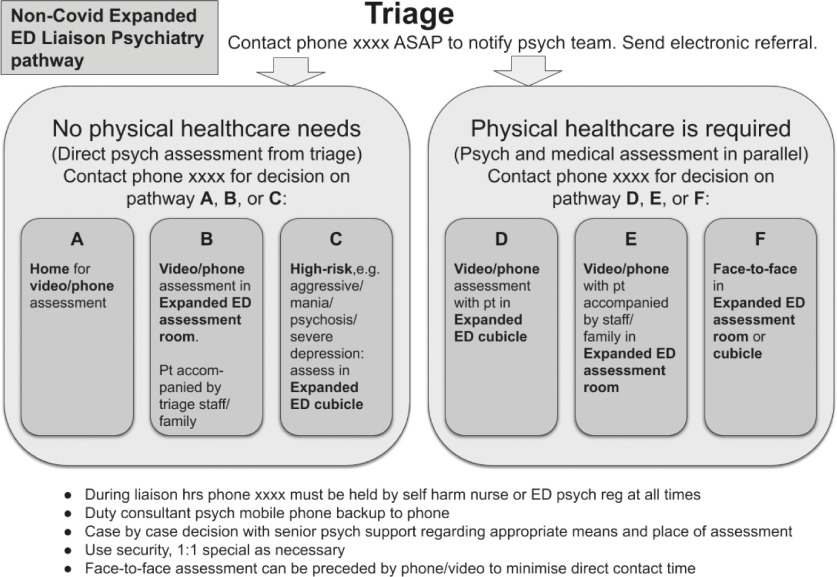
Beaumont Hospital COVID-19 ED patient assessment algorithm.

With the close collaboration of our ED colleagues, CLP has devised four patient pathways based on the separation of the ED into COVID-19 and Non-COVID-19 EDs and the expansion of the normal ED into an expanded ED taking over adjoining ward areas. These patient pathways were additionally designed to take into account the presence or absence of physical healthcare needs. The pathways have iterated on a frequent basis, as COVID-19 ED incrementally expanded and retracted into and from surrounding wards depending on daily numbers (see Figs. 2 and 3).

These new pathways utilise telepsychiatry (phone and video assessments) whenever possible for complete assessments, or initial assessment followed by a time-limited face-to-face assessment. Rapid assessment straight from triage was instigated for a suitable cohort of patients who had no physical healthcare needs, reducing time spent in ED and the number of unnecessary investigations as well as minimising patient and staff infection exposure time while still carrying out an emergency mental health assessment.

These pathways currently operate during the CLP daytime hours and some aspects (e.g., some telephone reviews but reverts to patients being seen first by ED medical staff prior to psychiatry review) are being implemented by the out of hours’ psychiatry service which is provided by the North Dublin Community Mental Health Service.

## University Hospital Waterford

The University Hospital Waterford is a 500-bed tertiary hospital which covers a catchment area of 500 000 in the south-east. The Department of Psychiatry in the University Hospital Waterford admits inpatients from the Counties of Waterford/Wexford/South Kilkenny, a mixed urban/rural catchment area of approximately 275 000 patients. There are on average over 1700 mental health presentations to the ED annually. The CLP team in UHW consists of 1 WTE Consultant, 1 WTE NCHD, 3 WTE Liaison CNSs. Prior to the COVID-19 situation, there were also 1 WTE Registered Advanced Nurse Practitioner in Psycho-Oncology and 1 WTE Registered Advanced Nurse Practitioner in Perinatal Mental Health – these have been both redeployed to the Community Diversion service – see below. At the time of submission in April 2020, the University Hospital Waterford has a low number of patients with confirmed COVID-19 cases compared to the national average. On April 13, there were 12 confirmed cases as inpatients in UHW.

Waterford/Wexford Mental Health Service has implemented an emergency plan to avoid exposure of the services’ patients to infection and to optimise the number of patients reviewed appropriately in the community.

## Community diversion

There is a single telephone number for the whole service operational 24 hours a day, 7 days a week which is answered by an experienced clinician (Registered Advanced Nurse Practitioner or CNS) and the call is diverted appropriately. This telephone number has been circulated to GPs in the south-east, the local out of hours GP on-call service, National Ambulance Service personnel and an Garda Siochana. Where possible, calls are forwarded to the appropriate CMHT who prioritise dealing with the patient in the community where possible unless it is an emergency.

## ED pathway

The psychiatry aspect of the ED in the University Hospital Waterford was functioning as usual (the ED in the University Hospital Waterford has not been divided into a COVID-19/non-COVID-19 pathway to date) as of 13 April. Shortly afterwards, UHW ED was divided into COVID-19/non-COVID-19 streams similar to the situation in other EDs nationally. The reason for the delay compared to other hospitals was that there were very low levels of confirmed COVID-19 cases in Waterford compared to the national average at that time. PPE is now being used in the COVID-19 ED pathway and is not being used in non-COVID-19 ED pathway.

A large perspex screen has been erected in the ED CLP interview room for the protection of liaison CNSs, on-call NCHDs and patients.

A separate new interview room has been commissioned adjacent to the Department of Psychiatry inpatient unit. This will be utilised in the event that the ED delineates into COVID-19/non-COVID-19 pathways. ED non-COVID-19 psychiatry presentations without an acute physical healthcare need (i.e., patients who are anxious, depressed, require advice around medications, suicidal ideation but no physical episode of self-harm, no evidence of agitation or delirium) will be diverted away from the ED after registering at ED triage and will be reviewed in this new separate assessment area.

Patients presenting to ED with mental health and comorbid acute physical healthcare needs or where there are concerns about acute agitation, aggression or serious risk will be reviewed in the ED CLP interview room as usual as per pre-COVID -19 protocol.

## SVUH

SVUH is part of the Ireland East Hospital network serving a primarily urban catchment area. The hospital has 536 inpatient beds and 78 day care beds and bed capacity increased by 44% (236 beds) in the 3-month private–public hospital agreement announced by the government on 30 March 2020. CLP assesses approximately 1700 patients in the ED annually constituting 3% of the total ED attendances. This is the only ED for three separate community mental healthcare sectors/approved centres and each sector represents approximately one-third of presentations. The CLP team in SVUH consists of one WTE Consultant, three WTE NCHDs and three WTE Liaison CNSs.

## ED pathway

**Fig. 4.**
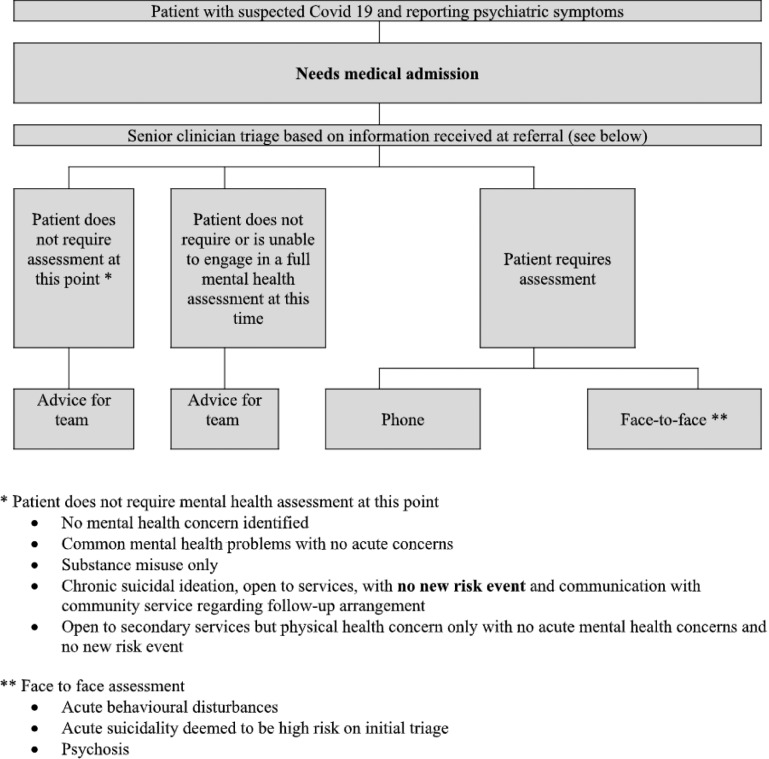
SVUH algorithm for patients requiring medical admission or already medically admitted.

**Fig. 5.**
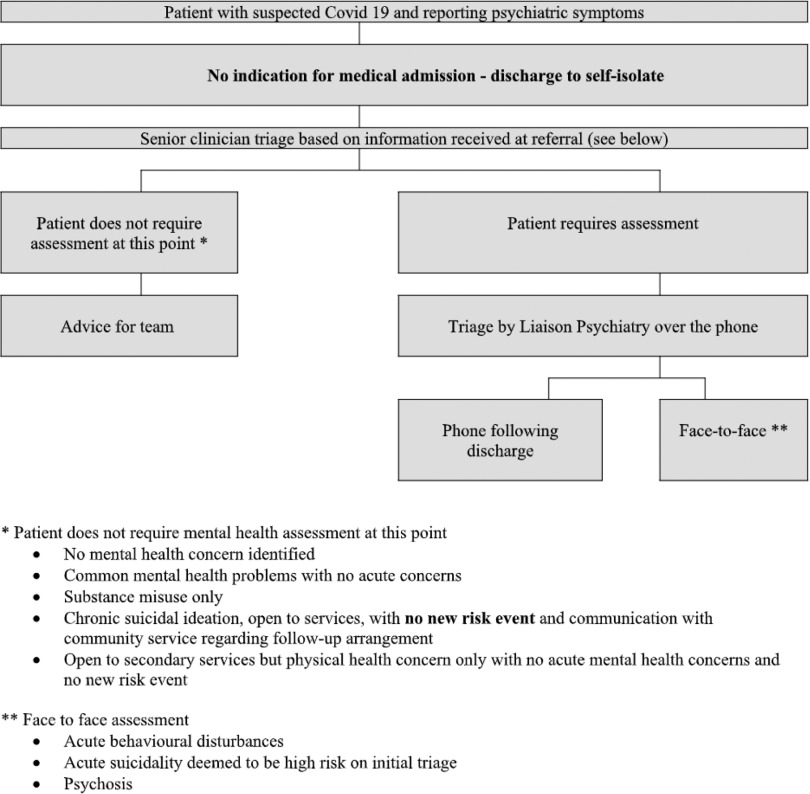
SVUH algorithm for patients that do not require medical admission.

The hospital including ED has been separated into non-COVID-19, suspected COVID-19 and COVID-19-positive zones. In conjunction with ED and acute medical assessment unit staff, we have formulated algorithms for patients with suspected COVID-19 in the ED with the priority being moving patients through the department as quickly as possible (see Figs. 4 and 5).

The criteria for patients that will likely require face-to-face assessment include acute behavioural disturbance, acute suicidality deemed to be high risk on initial triage or psychosis. Outside of this, we have found that phone assessments are not possible for some patients due to communication difficulties. We have found a combined phone/brief face-to-face assessment (<15 minutes) to be helpful in these cases.

Our frequent attender care plans (usually created for patients with chronic suicidal ideation, open to services, with no new risk event) have demonstrated their benefit in recent weeks, as attendance rates of some of these patients have anecdotally increased.

The solution we have arrived at relies on senior (consultant or senior registrar) triage of referrals either over the phone or face to face from 9 to 5 p.m. Senior decision-making and direct coordination of the CLP team has the advantage of expediting the development of management plans, particularly for known patients and reducing the length of stay. However, it has been onerous given the limited CLP team and is not sustainable in the longer term.

Patients in the ED who are not suspected of having COVID-19 and require both psychiatric and medical input are being transferred to the AMU to await investigations. We have assigned a CLP team member to the AMU to support staff there and provide a rapid assessment.

## Community diversion

We have been supported by our community mental health colleagues who have set up single points of contact for patients and general practitioners. They have provided urgent assessments (phone/face to face) following a brief assessment as described above in the ED. The AMU in St Columcille’s Hospital has been helpful in diverting psychiatric inpatients in the two approved centres not attached to SVUH, from the ED who require medical input.

## Discussion

The examples described above give a useful national perspective on the flexibility of our psychiatry services, both in CLP and General Adult Psychiatry (GAP) services, in implementing a variety of different solutions at a time of unprecedented challenges. While our examples provide a snapshot of CLP services, the limitations include the absence of paediatric CLP and the complete national picture. At the time of submission, data is not yet available regarding the impact of COVID-19 on ED presentations in Ireland and there is, to date, a lack of international data. The authors hope to track ED presentation numbers and submit for publication as they become available. The paper highlights the current high levels of activity within each of the CLP services, while also flagging the disparities in staffing levels (ranging from one to two and a half FTE CLPs), with none achieving the recommended Vision for Change staffing numbers of 1 liaison team per 500-bed general hospital comprised of 1 consultant, 1 doctor in training, 2 clinical psychologists, 5 CNSs and 2 administrative support staff. The paper demonstrates that at times of crisis, improvements to work practices can occur. Many CLP and GAP services have adapted to the need for physical distancing, and have utilised technological innovations to minimise face-to-face contact where possible: consistent with similar practice around the world in this pandemic (Whaibeh *et al*. 2020; Wind *et al*. 2020). Each service has implemented the use of PPE as per individual hospital guidelines, and in line with changes to guidelines as the situation and as information in relation to transmission of the virus evolved. At the time of submission, full PPE appropriate for non-aerosol-generating guidelines (HSE, 2020 PPE guidance for staff) is used for all COVID-19 suspected or confirmed patients and surgical masks are worn in all clinical settings irrespective of COVID-19 status while also implementing social distancing where possible.

CLP teams have been able to implement clearly defined, rapid pathways for patients with no physical healthcare needs; CMHTs have further developed effective community diversion pathways, all in keeping with the National Emergency Medicine Programme which states that the ED must not be the pathway of access to mental health care at any time of point in time for patients with mental ill-health who have no acute medical need (HSE, 2012). Patients attending mental health services, their families and advocacy groups have repeatedly called for alternatives to the ED as a site for emergency psychiatric assessment (O Feich, 2019). The need for alternative sites for assessment has become more clearly a patient-safety issue during this crisis, as minimising the exposure of patients with mental illness to infection is especially important considering that many will have comorbidities and may find the restrictions such as social/physical distancing more difficult (Lima *et al*. 2020; Xiang *et al*. 2020; Yao *et al.* 2020).

Additionally, there is considerable emerging evidence of post- COVID-19 psychiatric sequelae which will add to the already understaffed and under-resourced CLP services. Specifically, a cohort study of the first 153 diagnosed with COVID-19 in the UK found that 31% had altered mental status, of whom 41% had encephalopathy. The remaining 59% had another psychiatric diagnosis of whom 92% were new diagnoses; 43% with psychosis, 26% with a cognitive dementia-like syndrome and 17% an affective disorder (Varatharaj *et al.* 2020)

The Department of Health and Children (2006) recommended that each CMHT would provide a 24/7 multidisciplinary crisis intervention service, whilst noting that services tended to be determined by the resources available. In 1984, the mental health budget was 12.8% of the overall Irish health budget (O’Shea, 2008). In 2020, it is 6.04% of the overall health budget. This compares unfavourably to the average of 13% across EU countries in 2015 (OECD/EU, 2018). Despite these low resources and underfunding, in line with the rest of our medical colleagues, our psychiatry services have stepped forward to respond at a time of unprecedented challenge. We must not allow our patients to be left behind in a post-COVID-19 world. It is hoped that these clinical care improvements for patients with mental health needs will be sustained in the future and long beyond COVID-19.
